# Elevational changes in the bacterial community composition and potential functions in a Tibetan grassland

**DOI:** 10.3389/fmicb.2022.1028838

**Published:** 2022-11-10

**Authors:** Yaoming Li, Zhen Fang, Fan Yang, Baoming Ji, Xiangzhen Li, Shiping Wang

**Affiliations:** ^1^College of Grassland Science, Beijing Forestry University, Beijing, China; ^2^Key Laboratory of Environmental and Applied Microbiology, Chinese Academy of Sciences, and Environmental Microbiology Key Laboratory of Sichuan Province, Chengdu Institute of Biology, Chinese Academy of Sciences, Chengdu, Sichuan, China; ^3^Key Laboratory of Alpine Ecology, Institute of Tibetan Plateau Research, Chinese Academy of Sciences, Beijing, China; ^4^CAS Center for Excellence in Tibetan Plateau Earth Sciences, Beijing, China

**Keywords:** Tibetan plateau, soil bacterial community, potential function, alpine grassland, elevation gradient

## Abstract

In the Tibetan grasslands, the distribution patterns of the microbial community structure and function along elevation gradients have attracted considerable attention due to the wide distribution of mountain slopes, but the controlling factors of these patterns are still unclear. Here we investigated the taxonomy and potential functions of soil bacteria along an elevation gradient in a Tibetan mountainous grassland in 2 years, aiming to explore the elevation patterns of the bacterial structure and function and the underlying drivers. High-throughput sequencing and environment attribute measurements were conducted to survey the bacterial and environment characters. Furthermore, PICRUSt2 for prediction of bacterial functions, iCAMP for unraveling the drivers controlling community assembly, and HMSC for variance partitioning of bacterial community composition were used. Elevation did not significantly affect the bacterial diversity but changed their composition, driven by both deterministic and stochastic processes. In addition, elevation did not significantly affect the relative importance of deterministic and stochastic processes. Soil carbon, nitrogen, and temperature were important deterministic factors in driving bacterial community structure. The genus *Solirubrobacter* in *Actinobacteriota* was identified as most elevation discriminatory. Based on these observations, the bacterial community in the Tibetan mountainous grasslands was more controlled by edaphic factors than temperature, indicating their relative stability under climate change scenarios.

## Introduction

As the Third Pole on the earth, the Tibetan Plateau is a unique geographic unit with great biodiversity and critical ecosystem functions (Zhang et al., 2002; Qiu, 2008) and is experiencing striking climate warming (Duan et al., 2006; Qiu, 2008). In the last 50 years, the surface temperatures on the Tibetan plateau rose 1.5–1.8°C, approximately three times the global warming rate (Qiu, 2008).

Alpine grassland, covering more than 65% of the plateau area, is the largest ecosystem type on the Tibetan Plateau (Fu et al., 2021), and plays an important role in carbon sequestration (Schuman et al., 2002). Soil microbes are critical in terrestrial carbon cycling by controlling the balance between organic carbon decomposition and stabilization of microbial assimilated carbon (Garcia-Franco et al., 2015; Liang et al., 2017; Malik et al., 2020). Distinct microbial communities between lower and higher elevations has been reported on Tibetan mountains, and soil geochemical factors, such as pH value and nutrition conditions, were major factors affecting their structure and diversity along elevations (Xu et al., 2014; Wang et al., 2015; Shen et al., 2019). Although many studies have been conducted in Tibetan alpine grasslands, the generality of these distribution patterns and underlying mechanisms remain unclear (Cui et al., 2019). Understanding the mechanisms controlling community structure and their response to environmental change is a central, but long-standing challenge in microbial ecology (Stegen et al., 2013; Zhou and Ning, 2017). It has been well accepted that deterministic and stochastic processes simultaneously control microbial community assembly. However, their relative importance is still unclear in the Tibetan grasslands (Zhou and Ning, 2017; Ning et al., 2019, 2020). Edaphic and climate factors were reported as important selection stresses in microbial community assembly, resulting in different microbial response to climate change (Li et al., 2019). Thus, understanding the elevation patterns of bacterial structure and function and the underlying drivers is critical for projecting changes in soil carbon and improving the predictions of climate change feedbacks (Klein et al., 2004; Lin et al., 2009).

The rapid development of high-throughput sequencing technologies and analysis methods, such as PICRASt2 (Douglas et al., 2020) for the prediction of metagenome functions and iCAMP (Ning et al., 2020) for revealing the ecological drivers of microbial community assembly, have enabled scientists to conduct detailed investigations of microbial communities. In this study, we sampled soils from 4 elevations of a mountain slope on the Tibetan plateau in 2009 and 2015. Mountain slopes are important topographical features on the Tibetan Plateau and provide elevation/temperature gradients, which have been frequently used to reveal elevation patterns of microbial community (Li et al., 2016). We hypothesized that (1) the bacterial taxonomy and potential function structure differ among elevations, due to the differed soil physicochemical properties and climatic conditions; (2) the bacterial community assembly in higher elevations and colder years are more controlled by selection relative to that in lower elevations and warmer years, due to the lower nutrient content and temperature.

## Materials and methods

### Experimental site and soil sampling

The experimental site was described previously in detail (Wang et al., 2014a). In brief, the experiments were carried out in Haibei Alpine Meadow Ecosystem Research Station (37°37′N, 101°12′E) of the Northeastern Tibet Plateau, Qinghai, China. The local climate is typical plateau continental, characterized as warm and short in summer but cold and long in winter. The annual mean air temperature and precipitation from 1981 to 2000 are −1.7°C and 561 mm, respectively (Zhao et al., 2006). Soil type at the research sites is dominated by Mat Cryic Cambisols typical of alpine grassland soil (Yang et al., 2014). Four elevations of 3,200 m (37°36′42.3′′N, 101°18′47.9′′E), 3,400 m (37°39′55.1′′N, 101°19′52.7′′E), 3,600 m (37°41′46.0′′N, 101°21′33.4′′E), and 3,800 m (37°42′17.7′′N, 101°22′09.2′′E) were chosen for sampling. The geographic distances between adjacent elevations were 6.2 km (3,200 to 3,400 m), 4.2 km (3,400 to 3,600 m), and 1.3 km (3,600 to 3,800 m), respectively. The plant communities were significantly different among the four elevations. Plant communities in 3200 were dominated by *Kobresia humilis*, *Festucaovina*, and *Elymus nutans.* Plant communities in 3400 were dominated by alpine shrub *Potentilla fruticosa*, and jointly by *K. capillifolia*, *K. humilis*, and *Saussurea superba*. Plant communities in 3600 were dominated by *K. humilis*, *S. katochaete Maxim*, and *P.nivea*, and plant communities in 3800 were dominated by *K. humilis*, *L. odiumnanum*, and *Poa* spp. (Lin et al., 2009; Wang et al., 2014b).

One 20 m long × 8 m wide plot was fenced at each elevation location in 2006. At the center of each plot, HOBO weather stations (Onset Computer Corporation, Cape Cod, Massachusetts, United States) were used to monitor soil temperature at 5 cm depth and soil moisture at 20 cm depth. Data was collected at 1 min intervals, and 30 min averages were stored in the data logger.

Three 1 m × 1 m subplots were established within the plot in each elevation. Soil samples were collected in August 2009 and 2015, respectively. Five soil cores with a diameter of 3 cm at the depth of 0–10 cm were taken randomly from each subplot and mixed as one sample. In total, 24 soil samples (3 samples × 4 elevations × 2 years) were obtained. Then soil samples were transported back to the laboratory at 4°C in cooler boxes and sieved with a 2 mm mesh to remove visible grass roots and stones. Soil samples for pyrosequencing were kept at −80°C until DNA extraction, and soil samples for environmental variable measurements were stored at 4°C.

### Environmental variable measurements

Plant properties, including above-ground biomass, species richness, composition, and coverage, were measured simultaneously as soil sampling using established protocols (Wang et al., 2019). The plant species composition in 3600 plots were not recorded due to the partly disturbed by pika.

Soil biogeochemical variables were measured as previously described (Li et al., 2019). In brief, total carbon, total nitrogen, and total organic carbon were measured by a TOC-5000 A analyzer (Shimadzu Corp., Kyoto, Japan) and a Vario EL III Elemental Analyzer (Elementar, Hanau, Germany) using standard protocols (Ryba and Burgess, 2002). Soil NH_4_^+^-N and NO_3_^−^-N were analyzed with a FIAstar 5,000 Analyzer (FOSS, Hillerd, Danmark). The soil C/N ratio was calculated as the total carbon to total nitrogen ratio. Soil pH was measured by pH meter (HANNA HI 70004, HANNA Instruments, United States). Soil phosphate was extracted with Mehlich 3 solution and measured by inductively coupled plasma optical emission spectroscopy (ICP-OES) on an Agilent 5,110 ICP-OES (Agilent, United States).

### DNA extraction and sequencing

DNA extraction from soil samples, purification and pyrosequencing were described in previous studies (Wu et al., 2017). In brief, the Fast DNA Spin kit (MP Biomedical, Carlsbad, CA, United States) was used to extract DNA from 0.5 g soil, following the manufacturer’s instructions. The V4-V5 hypervariable regions of 16S rRNA genes were PCR amplified with primers 515F (5′-GTGCCAGCMGCCGCGGTAA-3′) and 907R (5′- CCGTCAATTCCTTTGAGTTT −3′). The detailed PCR and other experimental procedures were described by Li et al. (2019). The barcoded amplicons were pooled in an equimolar concentration and sequenced using a GS FLX system (454 Life Sciences, Branford, CT). All sequences were deposited in National Genomics Data Center (NGDC) (accession No. PRJCA011473).

### Data processing of pyrosequencing reads

Sequence quality control and amplicon sequence variant (ASV) table construction were conducted with DADA2 (Callahan et al., 2016), resulting in 221,341 high-quality and chimera-free reads (~370 bp). Then diversity and taxonomic analysis were conducted using Quantitative Insights Into Microbial Ecology version 2 (QIIME2) as described previously (Hall and Beiko, 2018). The ASV table was rarefied to a sequencing depth of 1,500 per sample for subsequent analyses of alpha and beta diversity. It has been reported that 1,500 denoised sequences per sample can explain more than 80 and 90% of the trends in α- and β-diversity, respectively, among samples observed for 15,000–20,000 bacterial sequences (Lundin et al., 2012). Thus, the rarified datasets should be acceptable when resampling to 1,500 denoised sequences here. Alpha diversity metrics (the number of observed ASVs, Shannon and Simpson index) and composition shifts were analyzed using the rarefied ASV table. To test the effects of elevation on bacterial composition, the samples from different elevations were clustered based on Bray-Curtis distance using R package vegan (Oksanen et al., 2013). Finally, we used the program PICRUSt2 (Phylogenetic Investigation of Communities by Reconstruction of Unobserved States) to predict the potential functional components of the microbiome (Douglas et al., 2020).

### Statistical analyses

All the following data analysis was performed with the R package vegan (Oksanen et al., 2013), iCAMP (Ning et al., 2020), randomForest (Liaw and Wiener, 2002), and HMSC (Tikhonov et al., 2020). Non-parametric multivariate analysis of variance (adonis) using Bray-Curtis distance matrices was used to test the microbial taxonomic and functional structure changes with elevation and experimental years. Two-way analysis of variance (ANOVA) was used to test the effects of elevation and experimental year on bacterial and environmental measurements. The random forest regression model was performed to identify key microbial ASVs associated with elevations and experimental years as previously described (Stewart et al., 2018). Infer community assembly mechanisms by phylogenetic bin-based null model analysis (iCAMP) and Hierarchical Modelling of Species Communities (HMSC) framework (Ovaskainen et al., 2017) were used to understand the community assembly processes controlling biodiversity patterns and their response to climate change. iCAMP was a robust and reliable tool for quantifying the relative importance of ecological processes in controlling microbial community assembly (Ning et al., 2020). HMSC is a general, flexible framework for modern analysis of community data, and it performs very well in explaining and predicting the occurrences of individual species or species assemblages (Ovaskainen et al., 2017). Thus, we used HMSC to partition observed variation in bacterial species occurrences into components related to environmental variation measured vs. random processes at the community level (Ovaskainen et al., 2017). Random processes here measure the variation in bacterial species occurrences and co-occurrences that cannot be attributed to the responses of the species to the measured environmental covariates, i.e., stochastic processes or unexplained variances.

## Results

### Soil physicochemical and plant measurements along the elevation gradients in 2 years

**Figure 1:**
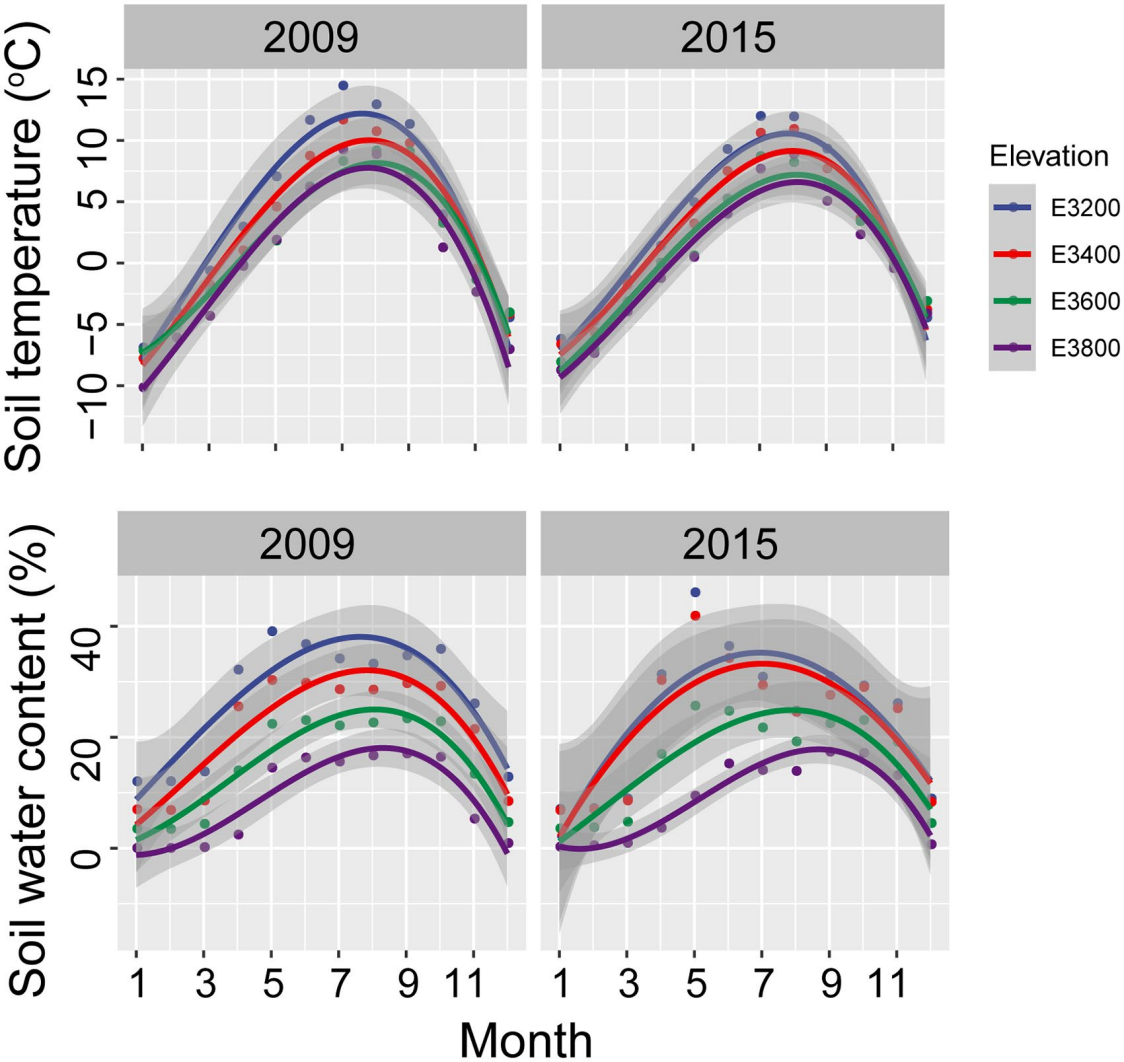
Monthly soil temperature at 5 cm and water content at 20 cm for various elevations in 2009 and 2015.

Soil temperature, water, and nutrient content decreased with increasing elevation (Supplementary Tables S1, S2). The annual soil temperature in 5 cm depth in 3200, 3,400, 3,600, and 3,800 were 3.96 ± 2.15°C, 2.58 ± 1.91, 1.48 ± 1.39, and, 0.36 ± 1.89°C, respectively, in 2009 (Figure 1). The corresponding soil water contents in 20 cm in 2009 were 26.88 ± 2.56, 21.14 ± 2.47, 14.96 ± 3.25, and 8.76 ± 2.12%, respectively (Figure 1). The annual temperatures for 3,200, 3,400, 3,600, and 3,800 sites in 2015 were 3.01 ± 1.90, 2.15 ± 1.75, 0.91 ± 1.68, and 0.21 ± 1.63°C, respectively. The corresponding soil water contents in 20 cm in 2015 were 23.98 ± 3.16, 22.72 ± 2.95, 15.78 ± 4.50, and 8.84 ± 2.25%, respectively (Figure 1). Averagely, the annual soil temperatures of the 4 elevations were 2.09 ± 1.41 and 1.57 ± 1.23°C in 2009 and 2015, respectively, indicating warmer conditions in 2009 relative to 2015. Better soil nutrient content and higher vegetative coverage, diversity, and aboveground net primary production were found in lower elevations relative to higher sites (Supplementary Tables S1, S2). Soil total C and N showed decreasing trend with increasing elevation in 2015 (Supplementary Tables S1, S2). Soil physicochemical properties showed a similar trend in 2009 as that in 2015, except for the higher annual soil temperature (Supplementary Table S2).

**Figure 2:**
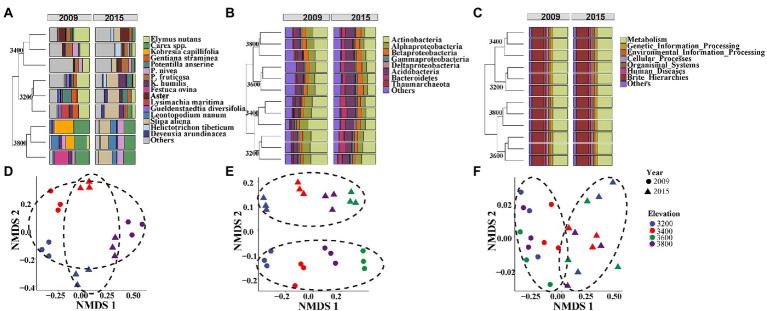
Composition of plant species **(A)**, bacterial phylum **(B)**, and potential bacterial functions **(C)** and their corresponding NMDS plots based on Bray-Curtis distance matrix in plant species compositions **(D)**, bacterial ASVs **(E)**, and potential genes **(F)** at four elevations in 2009 and 2015.

**Figure 3:**
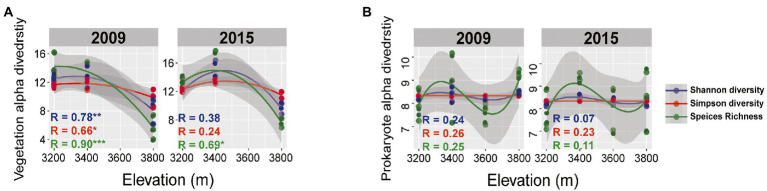
Plant **(A)** and bacterial alpha diversity **(B)** for various elevations in 2009 and 2015. Alpha diversity indexes were normalized to relative abundance. Gray area delineate 95% confidence intervals of the polynomial regression line. * means *p* < 0.05, ** means *p* < 0.01, and *** means *p* < 0.001.

Plant aboveground biomass and species richness decreased and composition changed with increasing elevation but remained unchanged between the 2 years (Supplementary Tables S1, S2). Plant composition was significantly different among the elevations and experimental years (Figures 2A,D; Table 1). At 3200 and 3,400, the plant community was dominated by *Elymus nutans* in 2009 (30 ± 1.63%), while *Stipa aliena* (32.7 ± 2.24%) and *Aster* (12.4 ± 5.03%) became the dominant specie in 3200 and 3,400 in 2015, respectively (Figure 2A). *Carex* spp. was dominant in 3800 in 2009 (34.97 ± 2.21%) and 2015 (28.54 ± 0.61%) (Figure 2A). Consistent with previous findings (Bryant et al., 2008), plant diversity was significantly changed with elevation and followed a decreasing pattern in 2009 and a unimodal pattern in 2015, respectively (Figure 3A; Table 2). The experiment year and its interactions with elevation did not show significant effects on plant diversity (Figure 3A; Table 2).

**Table 1:** Effects of elevation, experimental year, and their interactions on plant (Plant) and bacterial taxonomy (Bacteria taxonomy) and function (Bacterial Function) composition tested by Permutational Multivariate Analysis of Variance (ADONIS).

Source	Plant			Bacterial taxonomy			Bacterial Function		
	*df*	*R* ^2^	*p*	*df*	*R* ^2^	*p*	*df*	*R* ^2^	*p*
Elevation(E)	1	0.28	**0.001**	1	0.18	**0.001**	1	0.25	**0.001**
Year(Y)	1	0.20	**0.001**	1	0.13	**0.001**	1	0.33	**0.001**
E*Y	1	0.06	0.074	1	0.03	0.417	1	0.03	0.167

The degree of freedom (df) was 2 for plant species composition because plant data was not recorded in 3,600 plots due to the partly disturbed by pika. Bold values indicate significant effects at 0.05 level.

**Table 2:** Summary of two-way ANOVA test for the effects of experimental years, elevations, and their interaction of plant (Plant) and bacterial taxonomy (Bacteria taxonomy) and potential functional (Bacteria function) alpha diversity indexes.

α-Diversity	Source	Plant			Bacteria taxonomy			Bacteria function		
		*df*	*F*	*p*	*df*	*F*	*p*	*df*	*F*	*p*
Observed_species/genes	Year(Y)	1	1.934	0.186	1	26.266	**<0.001**	1	42.646	**<0.001**
	Elevation(E)	1	36.784	**<0.001**	1	2.427	0.135	1	0.908	0.352
	E*Y	1	2.096	0.17	1	0.931	0.346	1	0.495	0.49
Shannon diversity	Year(Y)	1	0.767	0.396	1	11.123	**0.003**	1	3.74	0.067
	Elevation(E)	1	13.902	**0.002**	1	1.176	0.291	1	10.949	**0.004**
	E*Y	1	1.051	0.323	1	1.026	0.323	1	2.332	0.142
Simpson diversity	Year(Y)	1	1.54	0.235	1	3.062	0.096	1	11.112	**0.003**
	Elevation(E)	1	8.991	**0.01**	1	0.505	0.486	1	2.973	0.1
	E*Y	1	1.469	0.246	1	0.456	0.508	1	1.731	0.203

Bold values indicate significant effects at 0.05 level.

### Distribution patterns of bacterial communities along the elevation gradient in 2 years

**Figure 4:**
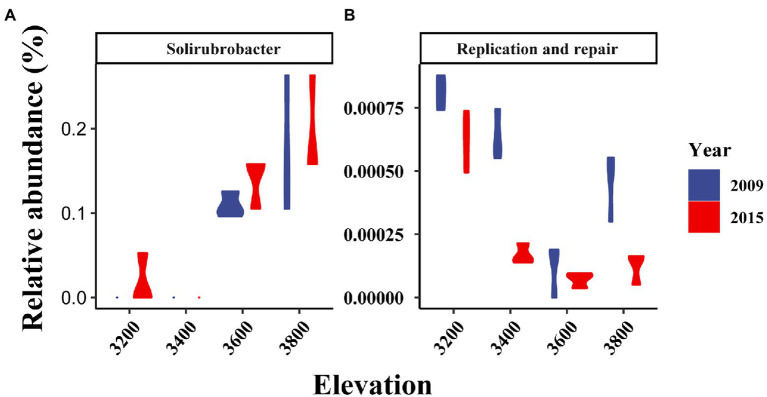
Changes in the relative abundance of *Solirubrobacter* **(A)** and potential replication and repair genes **(B)** with elevation in different years.

Bacterial taxonomic composition significantly changed along the elevation gradients (Table 1; Figures 2B,E). The bacterial communities were mostly represented by phyla *Actinobacteria* and *Proteobacteria*, which accounted for more than 50% of the reads in together (Figure 2B). The archaeal community was mostly represented by ammonia oxidizing genus *Nitrososphaera* in the phyla *Thaumarchaeota* at 2.4 ± 0.29% and 2.27 ± 0.17% in 2009 and 2015, respectively (Figure 2B). The relative abundance of *Actinobacteria* and *Thaumarchaeota* showed a pattern of first decreasing then increasing with elevation, while an opposite pattern was found for *Acidobacteria* and *Deltaproteobacteria* (Supplementary Figure S1). Twenty ASVs were identified as most elevation discriminatory over the 2 years by random forest regression analysis, half of which belonged to *Actinobacteria* (Supplementary Figure S2). An ASV belonging to the genus *Solirubrobacter* in *Actinobacteriota* was identified as most elevation discriminatory over the 2 years (Supplementary Figure S2). The relative abundance of the *Solirubrobacter* ASVs dramatically increased with increasing elevation (Figure 4A). Bacterial alpha diversity, including species richness, Shannon, and Simpson diversity were not significantly different with elevation (Table 2; Figure 3B).

In addition, the bacterial community composition changed significantly between the 2 years (Table 1; Figures 2B,E). For example, *Actinobacteria* was more enriched in 2009 (35.21 ± 4.17%) relative to 2015 (25.09 ± 3.68%), while *Acidobacteria* showed an opposite trend (Supplementary Figure S1). Bacterial alpha diversity, including species richness, Shannon, and Simpson diversity were significantly higher in 2015 relative to 2009 (Table 2; Figure 3B).

### Distribution patterns of potential bacterial function genes along the elevation gradient in 2 years

The composition of bacterial potential function was significantly affected by elevation and experimental year (Table 1; Figure 2F). The relative abundance of metabolism and organismal systems genes decreased with increasing elevations, while genetic information processing genes were enriched in higher elevations (Figure 2C). In addition, the relative abundance of metabolism, genetic information processing, environmental information processing, and cellular processes genes was significantly lower in 2015 relative to 2009 (Figure 2C). Most substrate utilization and nitrogen cycling genes decreased with increasing elevations (Supplementary Figure S3). For example, the potential functional genes in sugar utilization, cellobiose transport, cellulose, chitin, and hemicellulose degradation, and ammonification decreased with increasing elevation and their abundance was higher in 2015 compared with 2009 (Supplementary Figure S3). The potential methanogenesis genes increased with elevations (Supplementary Figure S3). Based on the random forest regression analysis, K18320 belonging to replication and repair functions was the most elevation discriminatory over the 2 years (Supplementary Figure S2b). The relative abundance of this gene dramatically decreased with increasing elevation (Figure 4B).

### Ecological processes controlling bacterial community assembly

**Figure 5:**
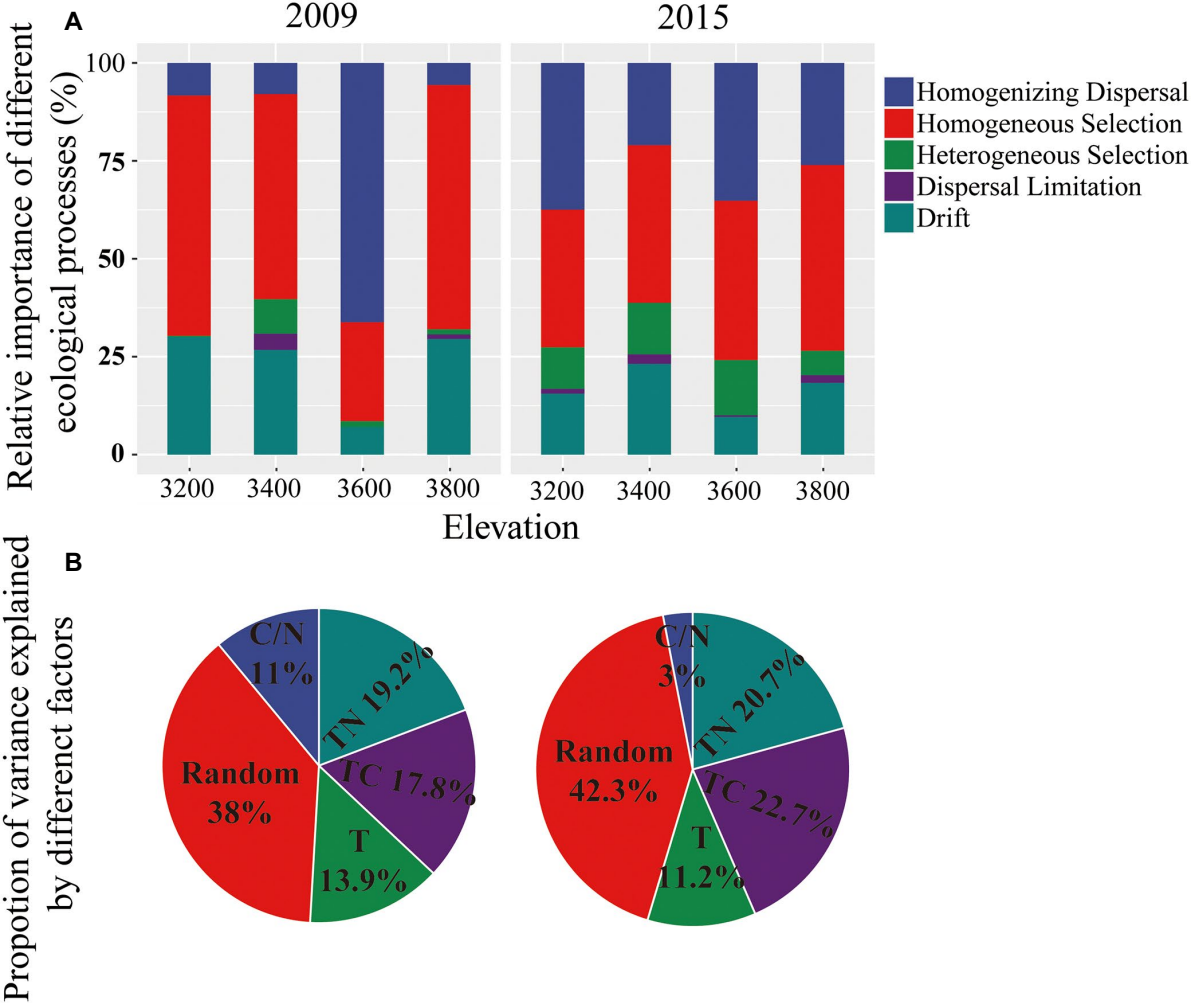
Relative importance of different ecological processes in controlling bacterial community composition for 4 elevations estimated with iCAMP **(A)** and variance partitioning of microbial community composition using HMSC statistical framework **(B)** in 2009 and 2015. TC, total organic carbon; TN, total nitrogen; C/N, carbon to nitrogen ratio; T, temperature.

The bacterial community assembly was controlled by both stochastic and deterministic processes and was not changed with elevation or experimental years. The relative importance of stochastic and deterministic processes in controlling bacterial community assembly were 46.7 ± 5.84% and 53.2 ± 5.85% in 2009, respectively (Figure 5A). The corresponding values in 2015 were 48.1 ± 2.4% and 51.9 ± 3.06%, respectively (Figure 5A). The importance of stochastic and deterministic processes was not changed with elevation or experimental year (Supplementary Table S3).

Homogenizing dispersal and drift were more important than other stochastic processes in bacterial community assembly, with an average relative importance of 22.04 ± 9.65% and 23.33 ± 3.63% in 2009, respectively. The homogeneous selection was the most important deterministic process, with an average relative importance of 50.32 ± 5.76% in 2009 (Figure 4A). The importance of each ecological process remained unchanged along elevations and years (Figure 4A; Supplementary Table S3).

### Contributions of environmental attributes to the deterministic processes in bacterial community assembly

To identify the strength of different environmental factors contributing to the deterministic processes in bacterial communities, we partitioned the community variations into components attributable to the environmental covariates vs. variation assigned to random effects using HMSC. Soil nutrient properties, that was carbon and nitrogen content, had a major influence on bacterial composition in both years, which together explained about half of the composition variances for the bacterial communities (Figure 5B). Temperature changes resulting from increasing elevations explained 13.9 ± 3.44% and 11.16 ± 2.13% of the variances in bacteria community composition in 2009 and 2015, respectively (Figure 5B). About 38 and 42.3% of the variances in bacteria community composition could be attributed to random processes or could not be explained by measured environmental factors in 2009 and 2015, respectively.

## Discussion

Bacterial taxonomic and potential functional composition were significantly changed with elevation and experimental year (Table 1). In addition, soil nutrients and temperature showed important influences on these changes (Figure 5B). These findings verified our first hypothesis that the bacterial taxonomic and potential functional structure differs among elevations and years, due to the differed soil and climatic conditions. The bacterial community was dominated by *Actinobacteria* here (Figure 2B), which well adapted to low temperatures by entering a dormant, metabolically quiescent but viable state (Taş et al., 2018). Soil total carbon and nitrogen were the most important factors influencing bacterial community composition (Figure 4B), which was well reported in previous studies (Yang et al., 2014; Xue et al., 2018). The soil total carbon and nitrogen decreased with elevation (Supplementary Table S1), which may benefit oligotrophic organisms, such as *Acidobacteria*. Cultivated members of *Acidobacteria* are known oligotrophs and respond negatively to increases in carbon (Eichorst et al., 2007). Thus, the increased relative abundance of *Acidobacteria* with increasing elevations here might be partly due to the decreased soil carbon content (Figure 1; Supplementary Figure S1). Furthermore, the decreases in the relative abundance of *Actinobacteria* may be due to the increase in *Acidobacteria*, as these groups likely share similar niches. The phylum *Thaumarchaeota* thus far comprises all known archaeal ammonia oxidizers (Pester et al., 2011), suggesting that this group may be an important component of ammonia oxidation. The potential carbon and nitrogen cycling genes decreased along elevation (Supplementary Figure S2), which might be the explanation for the lower soil respiration in higher elevations detected in our previous study (Hu et al., 2016a). In addition, we found the relative abundance of potential methanogenesis genes increased with elevations, which was in line with the lower CH_4_ uptake in higher elevations in our previous study (Hu et al., 2016b). The decreased relative abundance of potential methanogenesis genes might be due to two reasons: (1) the lack of substrate for methanogens, such as H_2_/CO_2_, acetate, formate, methanol, and methylamines. These substrates were produced from soil organic matter decomposition, which might decrease with increasing elevation indicated by decreasing microbial carbon and nitrogen cycling genes; (2) the reducing water content with increasing elevation (Supplementary Table S1) resulted in increasing aerobic conditions and consequently inhibited methanogenesis.

It is worth noting that, though temperature change along the elevations explained more than 10% of the variance of bacterial structure and potential function, their effects were still difficult to parse, due to the mixed effects of soil physicochemical properties (Figure 5). Thus, the changes in bacterial structure and potential function between the experiments years with similar soil physicochemical properties but different annual temperatures were used to verify the temperature effects. The changes in bacterial structure with increasing elevation or decreasing temperatures were generally supported by their changes between the 2 years with different annual temperatures. For example, the relative abundance of *Actinobacteria* were both enriched in lower elevations and warmer year (i.e., 2009) (Supplementary Figure S1). Similarly, *Acidobacteria* were enriched in higher elevations and colder year (i.e., 2015) (Supplementary Figure S1). However, the elevational patterns of bacterial potential function were not always consistent with yearly changes. For example, the relative abundance of sugar utilization, chitin degradation, and ammonification genes were lower in higher elevations, but were higher in colder years (i.e., 2015) (Supplementary Figure S3). These consistent results should be partly due to the mixed effects of soil physicochemical properties along elevations (Figure 5). *Solirubrobacter* species were abundant and classified as most elevation discriminatory over the 2 years by random forest regression analysis here (Supplementary Figure S2). *Solirubrobacter* species were often identified as keystone species in the bacterial co-occurrence networks in alpine grasslands (Li et al., 2015; Nunes et al., 2018; Jiang et al., 2021). Thus, *Solirubrobacter* species may serve as potential indicator bacteria in the Tibetan grasslands under climate change.

The elevational bacterial diversity pattern found here is inconsistent with previous findings, which found declines in microbial diversity with increasing elevation due to decreasing soil pH (Adamczyk et al., 2019; Shen et al., 2019). In our study, the soil pH did not significantly change with elevation (Supplementary Table S1), and might not be the dominant driver of the diversity pattern here. To understand the mechanisms of the elevational diversity pattern, we calculated the relative importance of drift and selection in controlling bacterial community assembly. Homogeneous selection and drift were identified as important deterministic and stochastic processes, respectively, in controlling bacterial community assembly (Figure 5). These findings are in line with the reports in the grasslands of the US Great Plains (Guo et al., 2018; Ning et al., 2020). The importance of homogeneous selection should be due to the environmental filtering based on species fitness differences, such as the low temperature in the Tibetan grassland. The low temperature on the Tibetan plateau should be a limiting factor for the plants and microbes revealed by HMSC (Figure 5B). The annual soil temperature of 3,200 was on average 3.49°C and decreased with increasing elevation (Figure 1), which was dramatically lower than the optimum temperature of vegetation and bacterial growth in the Qinghai-Tibet Plateau (i.e., 7°C) (Cui, 2013).

However, our second hypothesis, namely that the bacterial community assembly in higher elevations and colder years are more controlled by selection relative to that in lower elevations/warmer year was not supported. The relative abundance of deterministic and stochastic processes in controlling bacterial community assembly remained unchanged with elevation and experimental years (Figure 5A; Supplementary Table S3). The lower temperature and nutrient content in higher elevations pose selection pressure (Figure 5B), but may simultaneously stimulate positive associations among bacterial species (Dai et al., 2022), which increase stochastic processes (Huang et al., 2015). Thus, the unchanged community assembly mechanisms should be partly due to the balance between the physicochemical selection pressure and positive interactions among bacterial species. In addition, the relative stability of soil pH along the elevations may also contribute to the unchanged community assembly mechanisms (Supplementary Table S1). It has been found that soil pH rather than elevation determines bacterial community assembly on Mt. Norikura, Japan (Cho et al., 2019).

## Conclusion

The bacterial taxonomic composition changed significantly with increasing elevation and years with different annual temperatures, potentially a result of the changed soil nutrient contents and temperature. *Solirubrobacter* in *Actinobacteriota* was identified as the most elevation discriminatory over the 2 years. Both deterministic and stochastic processes play important roles in controlling soil bacterial community assembly in this Tibetan grassland, and they were not changed with elevation or experimental years. In summary, these findings indicate the relative stability of the soil bacterial community under warming scenarios.

## Data availability statement

The data presented in the study have been deposited in the National Genomics Data Center (NGDC, https://ngdc.cncb.ac.cn/), accession number PRJCA011473.

## Author contributions

YL and SW designed the study. YL, ZF, FY, and BJ collected field data and samples for the study. YL, ZF, and XL conducted the sequencing of the samples. YL and BJ analyzed the data and wrote the manuscript. All authors contributed to the final version of the manuscript.

## Funding

This work was supported by the Fundamental Research Funds for the Central Universities (BLX201939), the National Natural Science Foundation of China (41871067 and 31872994), and the Second Tibetan Plateau Scientific Expedition and Research (STEP) Program (2019QZKK0302).

## Conflict of interest

The authors declare that the research was conducted in the absence of any commercial or financial relationships that could be construed as a potential conflict of interest.

## Publisher’s note

All claims expressed in this article are solely those of the authors and do not necessarily represent those of their affiliated organizations, or those of the publisher, the editors and the reviewers. Any product that may be evaluated in this article, or claim that may be made by its manufacturer, is not guaranteed or endorsed by the publisher.
