# Supplementary material for: Elevational changes in the bacterial community composition and potential functions in a Tibetan grassland
Source: Front Microbiol. 2022 Nov 10;13:1028838. doi: 10.3389/fmicb.2022.1028838 (PMC9684203; doi:10.3389/fmicb.2022.1028838)
Supplement: Supplementary file 1 [file Data_Sheet_1.docx]

Table S1. Soil physiochemical and plant properties of the experimental groups of the study in 2015. Results of plant measurements at 3600m elevation are not shown due to the disturbance by pika.

| Variables | 3200 | 3400 | 3600 | 3800 |
| --- | --- | --- | --- | --- |
|  |  |  |  |  |
| Total phosphorus (%) | 0.057±0.021 | 0.086±0.001 | 0.096±0.001 | 0.086±0.002 |
| Available phosphorus (mg/kg) | 0.01±0.003 | 0.003±0.001 | 0.004±0 | 0.005±0 |
| TOC (%) | 7.313±0.066 | 6.33±0.144 | 7.418±0.025 | 5.011±2.085 |
| NH_4_^+^-N (mg/kg) | 34.56±3.797 | 38.593±6.928 | 21.763±3.52 | 28.093±6.869 |
| NO_3_^-^-N (mg/kg) | 9.278±3.727 | 12.73±3.6 | 8.853±1.35 | 11.588±0.347 |
| pH | 6.82±0.049 | 6.81±0.021 | 6.92±0.059 | 7.183±0.243 |
| Total N (%) | 0.842±0.028 | 0.605±0.022 | 0.795±0.048 | 0.673±0.031 |
| Total C (%) | 9.29±0.38 | 6.752±0.215 | 9.447±0.533 | 7.782±0.466 |
| C:N ratio | 11.024±0.122 | 11.176±0.232 | 11.895±0.072 | 11.554±0.167 |
| Plant coverage (%) | 100 | 100 |  | 93.5±0.289 |
| Plant species richness | 21.333±0.333 | 24.333±2.404 |  | 13±0.577 |
| Plant Shannon diversity | 2.055±0.034 | 2.609±0.143 |  | 1.716±0.105 |
| Plant above-ground biomass (g/m^2^) | 559.297±15.284 | 429.601±40.368 |  | 201.684±4.128 |
| Annual soil temperature | 3.01 | 2.15 | 0.91 | 0.21 |
| Soil volumetric water content (%) | 23.98±3.16 | 22.72±2.95 | 15.78±4.50 | 8.84±2.25 |

Values are given as the mean ± the SEM. SEM were not shown if they were less than 0.001.

Table S2 Results of two-way analysis of variance (ANOVA) for effects of elevation and experimental year on soil and plant measurements. Significantly changed measurements by elevation or experimental year were shown in bold.

| Soil and plant measurements | Source | Df | F | p |
| --- | --- | --- | --- | --- |
| NO_3_^-^-N | Year(Y) | 1 | 12.634 | **0.025** |
|  | Elevation(E) | 1 | 0.184 | 0.673 |
|  | Y*E | 1 | 0.009 | 0.924 |
| Total N | Year(Y) | 1 | 0.309 | 0.584 |
|  | Elevation(E) | 1 | 5.451 | **0.026** |
|  | Y*E | 1 | 0.760 | 0.239 |
| Total C | Year(Y) | 1 | 0.067 | 0.799 |
|  | Elevation(E) | 1 | 5.013 | **0.037** |
|  | Y*E | 1 | 2.629 | 0.121 |
| Plant coverage | Year(Y) | 1 | 0.433 | 0.518 |
|  | Elevation(E) | 1 | 15.244 | **0.033** |
|  | Y*E | 1 | 1.314 | 0.265 |
| Plant species richness | Year(Y) | 1 | 1.054 | 0.102 |
|  | Elevation(E) | 1 | 23.241 | 0.087 |
|  | Y*E | 1 | 5.110 | 0.372 |
| Plant Shannon diversity | Year(Y) | 1 | 3.007 | 0.134 |
|  | Elevation(E) | 1 | 12.882 | **0.023** |
|  | Y*E | 1 | 2.960 | **0.041** |
| Plant above-ground biomass | Year(Y) | 1 | 2.070 | 0.270 |
|  | Elevation(E) | 1 | 11.057 | **0.032** |
|  | Y*E | 1 | 0.510 | 0.824 |
| Annual soil temperature | Year(Y) | 1 | 9.010 | **0.049** |
|  | Elevation(E) | 1 | 68.548 | **0.001** |
|  | Y*E | 1 | 1.070 | 0.056 |

Table S3 Results of two-way analysis of variance (ANOVA) for effects of elevation and experimental year on ecological processes in controlling soil bacterial assembly.

| Ecological processes | Source | Df | F | p |
| --- | --- | --- | --- | --- |
| Deterministic processes | Year(Y) | 1 | 0.045 | 0.836 |
|  | Elevation(E) | 1 | 0.003 | 0.958 |
|  | Y*E | 1 | 0.816 | 0.384 |
| Homogeneous Selection | Year(Y) | 1 | 2.293 | 0.156 |
|  | Elevation(E) | 1 | 0.056 | 0.817 |
|  | Y*E | 1 | 1.209 | 0.293 |
| Heterogeneous Selection | Year(Y) | 1 | 3.89 | 0.072 |
|  | Elevation(E) | 1 | 0.201 | 0.661 |
|  | Y*E | 1 | 0.048 | 0.830 |
| Stochastic processes | Year(Y) | 1 | 0.045 | 0.836 |
|  | Elevation(E) | 1 | 0.003 | 0.958 |
|  | Y*E | 1 | 0.816 | 0.384 |
| Homogenizing Dispersal | Year(Y) | 1 | 0.558 | 0.47 |
|  | Elevation(E) | 1 | 0.102 | 0.755 |
|  | Y*E | 1 | 0.55 | 0.473 |
| Dispersal Limitation | Year(Y) | 1 | 0.053 | 0.821 |
|  | Elevation(E) | 1 | 0.007 | 0.935 |
|  | Y*E | 1 | 0.008 | 0.929 |
| Drift | Year(Y) | 1 | 2.326 | 0.153 |
|  | Elevation(E) | 1 | 0.455 | 0.513 |
|  | Y*E | 1 | 0.169 | 0.688 |


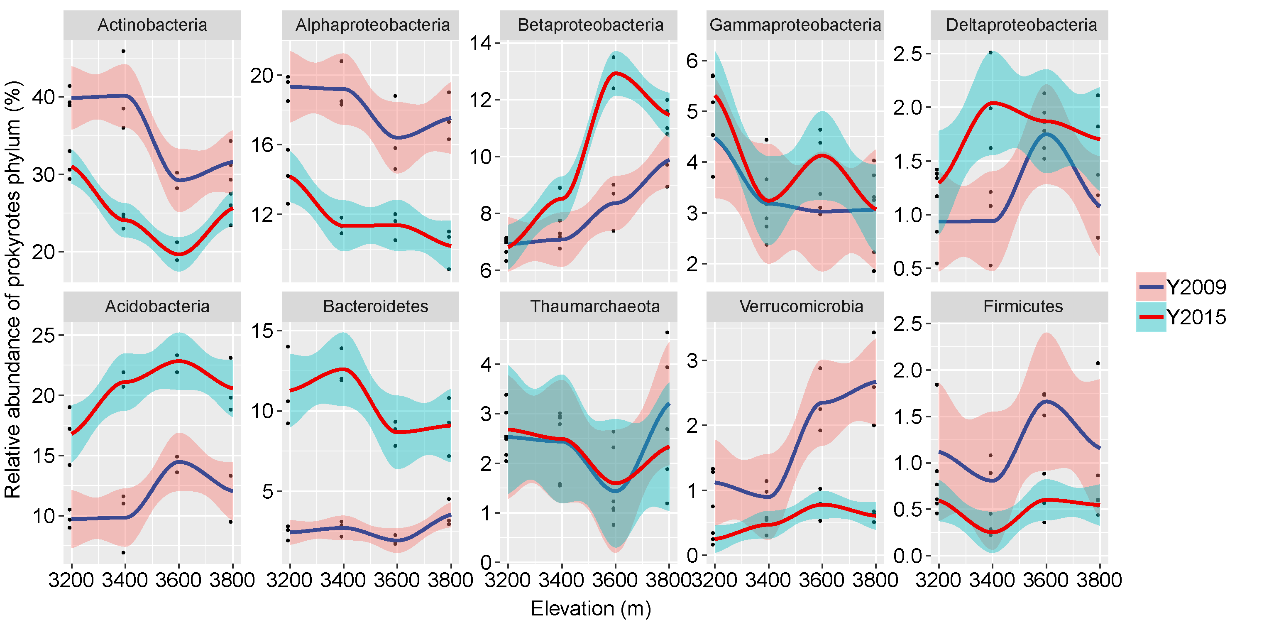


Figure S1 Changes in relative abundance of bacterial phylum with elevation in different years.


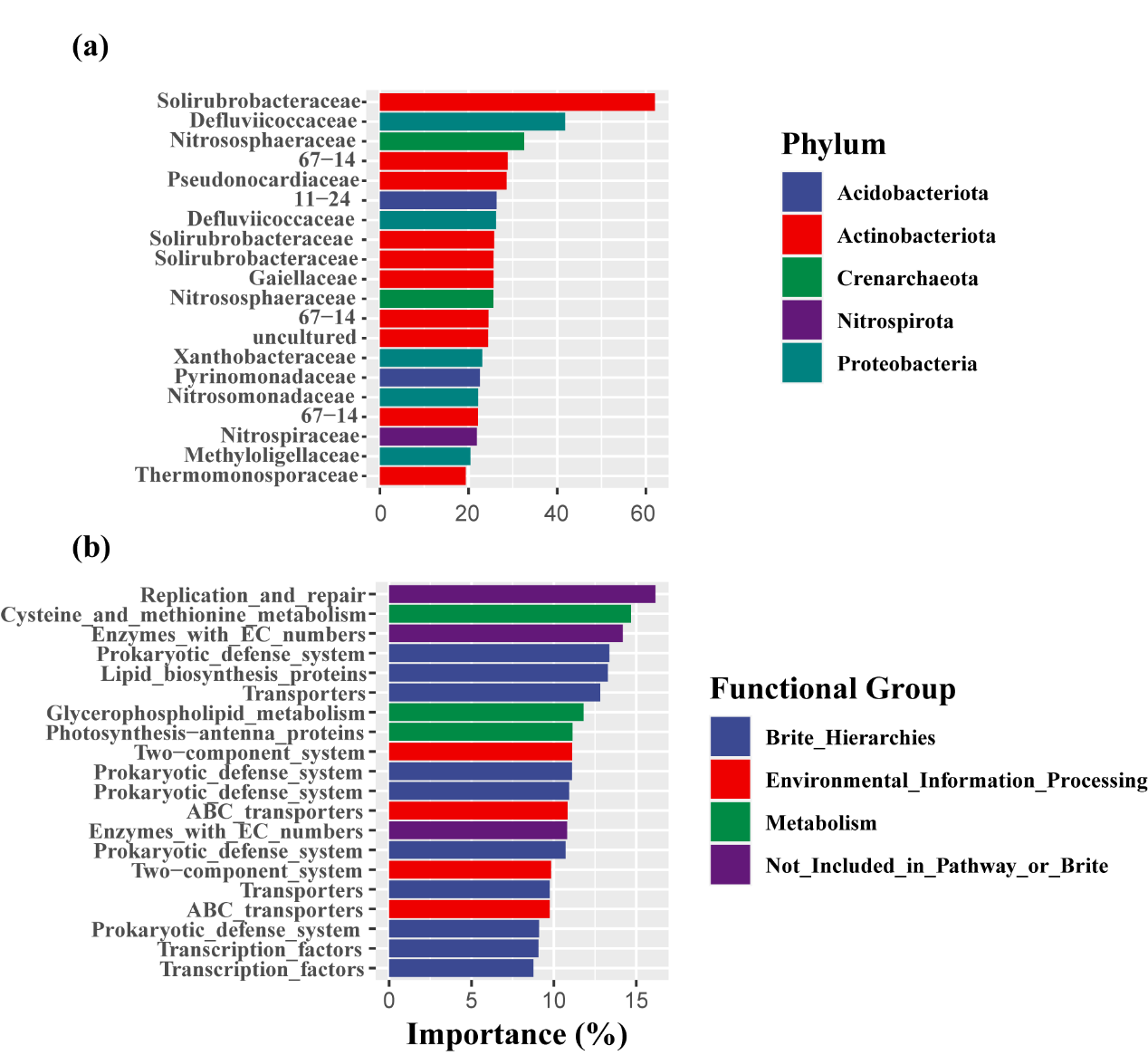


Figure S2. Twenty bacterial ASVs (a) and potential functional genes (b) classified by random forest regression analysis as most elevation discriminatory over the two years. The importance of ASVs and potential functional genes was determined by percent mean decrease in Gini. ASVs were named to the family level.


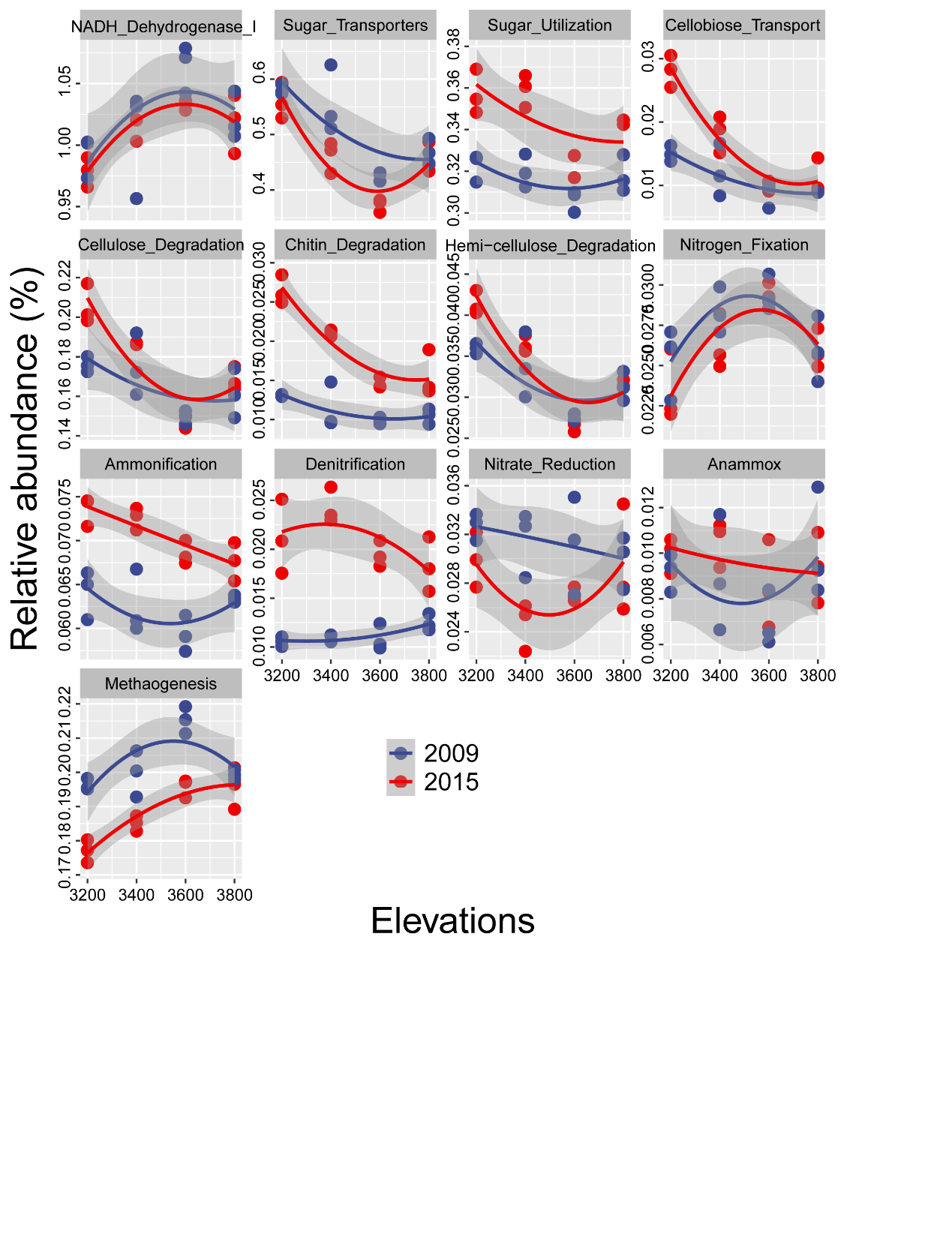


Figure S3 Changes in relative abundance of potential carbon and nitrogen cycling genes with elevation in different years.
